# Antimicrobial treatment duration for uncomplicated bloodstream infections in critically ill children: a multicentre observational study

**DOI:** 10.1186/s12887-022-03219-z

**Published:** 2022-04-05

**Authors:** Sandra Pong, Robert A. Fowler, Srinivas Murthy, Jeffrey M. Pernica, Elaine Gilfoyle, Patricia Fontela, Asgar H. Rishu, Nicholas Mitsakakis, James S. Hutchison, Michelle Science, Winnie Seto, Philippe Jouvet, Nick Daneman, on behalf of the Canadian Critical Care Trials Group

**Affiliations:** 1 Department of Pharmacy, The Hospital for Sick Children, Toronto, ON Canada; 2 Interdepartmental Division of Critical Care Medicine, University of Toronto, Toronto, ON Canada; 3 Tory Trauma Program, Sunnybrook Health Sciences Centre, Toronto, ON Canada; 4 Institute of Health Policy, Management and Evaluation, University of Toronto, Toronto, ON Canada; 5 Department of Pediatrics, Division of Critical Care, University of British Columbia, Vancouver, BC Canada; 6 Research Institute, BC Children’s Hospital, Vancouver, BC Canada; 7 Division of Infectious Diseases, McMaster University, Hamilton, ON Canada; 8 Department of Critical Care Medicine, The Hospital for Sick Children, Toronto, ON Canada; 9 Department of Epidemiology, Biostatistics and Occupational Health, McGill University, Montreal, QC Canada; 10 Department of Pediatrics, McGill University, Montreal, QC Canada; 11 Institute for Clinical Evaluative Sciences, Sunnybrook Health Sciences Centre, Toronto, ON Canada; 12 Children’s Hospital of Eastern Ontario Research Institute, Ottawa, ON Canada; 13 Dalla Lana School of Public Health, University of Toronto, Toronto, ON Canada; 14 Division of Infectious Diseases, Department of Paediatric Medicine, The Hospital for Children, Toronto, ON Canada; 15 Leslie Dan Faculty of Pharmacy, University of Toronto, Toronto, ON Canada; 16 Pediatric Intensive Care Unit, Sainte-Justine Hospital University Center, Montreal, QC Canada; 17 Department of Pediatrics, Université de Montréal, Montreal, QC Canada; 18 Division of Infectious Diseases, Sunnybrook Health Sciences Centre, Toronto, ON Canada

**Keywords:** Antibacterial agents, Bacteremia, Critical care, Critical illness, Duration of therapy, Pediatric

## Abstract

**Background:**

Bloodstream infections (BSIs) cause significant morbidity and mortality in critically ill children but treatment duration is understudied. We describe the durations of antimicrobial treatment that critically ill children receive and explore factors associated with treatment duration.

**Methods:**

We conducted a retrospective observational cohort study in six pediatric intensive care units (PICUs) across Canada. Associations between treatment duration and patient-, infection- and pathogen-related characteristics were explored using multivariable regression analyses.

**Results:**

Among 187 critically ill children with BSIs, the median duration of antimicrobial treatment was 15 (IQR 11–25) days. Median treatment durations were longer than two weeks for all subjects with known sources of infection: catheter-related 16 (IQR 11–24), respiratory 15 (IQR 11–26), intra-abdominal 20 (IQR 14–26), skin/soft tissue 17 (IQR 15–33), urinary 17 (IQR 15–35), central nervous system 33 (IQR 15–46) and other sources 29.5 (IQR 15–55) days. When sources of infection were unclear, the median duration was 13 (IQR 10–16) days. Treatment durations varied widely within and across PICUs. In multivariable linear regression, longer treatment durations were associated with severity of illness (+ 0.4 days longer [95% confidence interval (CI), 0.1 to 0.7, *p* = 0.007] per unit increase in PRISM-IV) and central nervous system infection (+ 17 days [95% CI, 6.7 to 27.4], *p* = 0.001). Age and pathogen type were not associated with treatment duration.

**Conclusions:**

Most critically ill children with BSIs received at least two weeks of antimicrobial treatment. Further study is needed to determine whether shorter duration therapy would be effective for selected critically ill children.

**Supplementary Information:**

The online version contains supplementary material available at 10.1186/s12887-022-03219-z.

## Background

Bloodstream infections (BSIs) are associated with significant morbidity and mortality in critically ill pediatric patients. A large Canadian study reported an overall annual BSI incidence of 53.7 per 100,000 among children [1], while a retrospective cohort study of hospitalized children in the United States found that patients with BSIs had significantly longer hospital stays (median 10 days vs. 2 days), more admissions to the intensive care unit (46% vs. 10%) and higher crude in-hospital mortality (5% vs. 0.34%) compared to non-BSI patients [2]. While early administration of adequate antimicrobial therapy has generally been found to be associated with decreased mortality in patients with infections [3–8], the optimal antimicrobial treatment duration for BSIs is understudied [9, 10]. Most treatment recommendations are based on limited studies, expert opinions and clinical experience [11, 12]. Consequently, there remains a research gap as to whether there are patients for whom shorter antimicrobial treatment durations would be adequate to achieve clinical cure while avoiding adverse drug reactions and toxicities, emergence of antimicrobial resistance, and increased healthcare-related costs associated with excessive treatment durations [9, 10, 13, 14].

Despite current research and experiences with antibiotic treatment duration for BSIs in adults [15–18], it cannot be assumed that these data can be directly extrapolated to infants and children. Pediatric patients have different underlying diseases, comorbidity patterns, age-related susceptibilities and exposures to certain infections. The microbiologic etiology of their BSIs, patterns of antimicrobial resistance and clinical outcomes are distinct from adults [19–22]. The pharmacokinetics and pharmacodynamics of antimicrobials are also different in young infants, who are at the highest risk of bloodstream infections [23, 24]. Studies in critically ill children are needed to produce evidence and guide treatment strategies that will optimize BSI outcomes and minimize treatment harms.

The objectives of this multicentre, retrospective observational study were to describe prevailing approaches of antimicrobial treatment duration for BSIs in critically ill pediatric patients and to explore associations between treatment duration and patient-, infection- and pathogen-related characteristics. We hypothesized that there would be wide variation in the durations of antibiotics used to treat BSIs in critically ill infants and children, and the median treatment duration would be similar or longer than those used in adults [15]. We also hypothesized that patients’ age, severity of illness, existing comorbidities, pathogen type and sources of infection would have an influence on the duration of antimicrobial therapy received.

## Methods

### Study design and setting

We conducted a retrospective observational cohort study of pediatric patients with BSIs in six pediatric intensive care units (PICUs) across Canada. Institutional research ethics board approval with waiver for informed consent was obtained at all sites.

### Patient selection

We included all patients younger than 18 years of age who had a blood culture yielding pathogenic bacteria and/or fungi that was reported positive between January 2015 and December 2017, while admitted to a PICU. We excluded patients with single positive blood cultures representing probable contamination (coagulase negative staphylococci, *Bacillus* species, *Corynebacterium* species, *Cutibacterium* species, *Aerococcus* species, *Micrococcus* species), patients with complicated BSIs where need for prolonged treatment is more established (infective endocarditis, osteomyelitis, septic arthritis, undrainable abscess, unremovable prosthetic material) [15, 25–28], patients who did not receive any antimicrobial therapy and patients previously enrolled in the study.

### Data collection

Data were collected by one of the authors (SP), experienced research coordinators and supervised medical and pharmacy students. A web-based electronic case report form derived from a similar form previously used to study antimicrobial treatment duration in critically ill adult patients was modified for use in pediatric patients and utilized to record data [15]. The case report form provided initial automatic checks for missing and invalid data. One of the authors (SP) performed a manual validation of the electronic data afterwards. Patient demographics, reasons for admission, severity of illness, comorbidities, source of BSI, pathogens and susceptibilities, antimicrobial treatments, and outcomes (relapse of infection, PICU readmission, mortality status at discharge from PICU and hospital) were collected. Patients were considered immunosuppressed if they had one or a combination of: hematopoietic stem cell transplant, malignancy, solid organ transplant, neutropenia, systemic corticosteroids, chemotherapy, asplenia or immunodeficiency syndrome.

### Duration of adequate antimicrobial treatment

The duration of adequate antimicrobial treatment was defined as the number of consecutive days on or after blood culture collection during which the patient received an antimicrobial regimen to which the index blood culture isolate(s) was/were all susceptible. The total duration could extend as far back as the date of the initial blood culture collection and could include multiple adjacent or overlapping courses of different antimicrobials to which the pathogens were susceptible. To allow for clinical scenarios where dosing intervals may be extended intentionally, such as renal impairment and delayed drug clearance, a maximum of one day was allowed during the treatment course that the patient did not receive at least one effective antimicrobial agent [15].

### Statistical analyses

Patient characteristics were summarized using descriptive statistics (frequencies with percentages, means with standard deviation [SD] and medians with interquartile range [IQR]). The primary outcome was the duration of adequate antimicrobial treatment, summarized with descriptive statistics (median [IQR]) for the overall cohort, for each participating PICU and for subgroups stratified by age, severity of illness, comorbidities, sources of infection and pathogens. Median treatment durations according to patient and pathogen variables were compared using Wilcoxon rank sum test for continuous variables (for 2 groups) and Kruskal–Wallis test (for > 2 groups). Patients who died within 10 days of their BSI and were still receiving antimicrobials were excluded from analyses to mitigate survivor bias because their antimicrobial treatments were discontinued due to death instead of clinical decision-making.

Multivariable linear regression with a random effect for PICU site to account for patient clustering, was used to examine the relationship between treatment duration and independent variables identified a priori based on clinical relevance. These variables were included in the regression model regardless of their statistical significance in univariate analyses: patient age, severity of illness (PRISM-IV score), comorbidities, pathogen and source of infection.

Sensitivity analyses of median treatment duration and regression analyses were performed by excluding patients with fungemia, patients less than 3 months old and patients with central nervous system (CNS) infections. We also performed a separate multivariable linear regression adjusted for PICU site to determine the effect of study site on the regression model.

All comparisons were two-sided and *p*-value < 0.05 was considered statistically significant. No adjustments were made for multiple comparisons. Statistical analyses were conducted using SAS statistical software version 9.4M6 (SAS Institute, Cary, NC) and R version 4.0.2.

### Sample size

For the primary objective, we required data from 196 patients to estimate treatment duration to within ± 7% precision for a 95% confidence interval, or 151 patients for an estimate within ± 8% precision. This was based on our hypothesis that critically ill children would have a median treatment duration for BSIs that is similar or greater than 14 days, as previously reported in critically ill adults [15]. To avoid over-representation of any single PICU, we allowed for a maximum of 75 patients per PICU site so that no one PICU would comprise more than half of the minimum required sample size.

## Results

### Patient and pathogen characteristics

The study population included a total of 211 critically ill children with BSIs in six PICUs across four provinces in Canada. For analyses of prescribed treatment duration, 24 patients were excluded because they died within 10 days of initiation of antimicrobials while still receiving therapy (*n* = 8) or had missing data regarding antimicrobial treatments or organisms (*n* = 16). Table 1 summarizes the baseline demographic data of the remaining 187 patients in the study cohort. The median age of the patients was 0.75 (IQR 0.25–4) years, 54% were male and 49% had medical indications for admission to the PICU. The most common reasons for admission were respiratory distress and septic shock.

**Table 1:** Characteristics of critically ill children with bloodstream infections

Characteristic	*n* = 187
Age, years	
Median (IQR)	0.75 (0.25–4)
Male sex, n (%)	100 (54)
Admission category, n (%)	
Medical	91 (49)
Cardiac-surgical	44 (24)
Surgical	22 (12)
Neurological/neurosurgical	5 (3)
Trauma	3 (2)
Burns	3 (2)
Other/missing	19 (10)
Reasons for ICU admission, n (%)^*a*^	
Respiratory distress	45 (24)
Septic shock	45 (24)
Surgery	37 (20)
Congenital heart disease	28 (15)
Bloodstream infection	14 (8)
Pneumonia	12 (6)
Other	101 (54)
PRISM-IV	
Mean (SD)	8.4 (7.2)
Median (IQR)	8 (3–12)

*SD* standard deviation, *IQR* interquartile range

^*a*^Total does not equal 187 because patients could have multiple reasons for PICU admission

The most common comorbidities found among patients were cardiovascular, respiratory, neurological and immunosuppression. There was a total of 225 organisms isolated from index blood cultures among the 187 patients included in the study–156 patients grew one organism, 26 patients grew two organisms, and 5 patients grew three or more organisms. Among 179 patients for whom source control procedure information was available, 105 (58.7%) did not undergo any source control measures and the remaining had one or a combination of the following: 60 (33.5%) had central vascular catheters removed or exchanged, 10 (5.6%) underwent surgical interventions involving the thorax or abdominal cavity, 7 (3.9%) had abscesses drained, 4 (2.2%) underwent chest tube insertions and 3 (1.7%) wound debridements and 5 (8.3%) had other procedures involving relief of ureter/bladder obstruction, Foley catheter removal/exchange, extracorporeal membrane oxygenation circuit change and pigtail insertion (unspecified site).

### Duration of adequate antimicrobial treatment

**Fig. 1:**
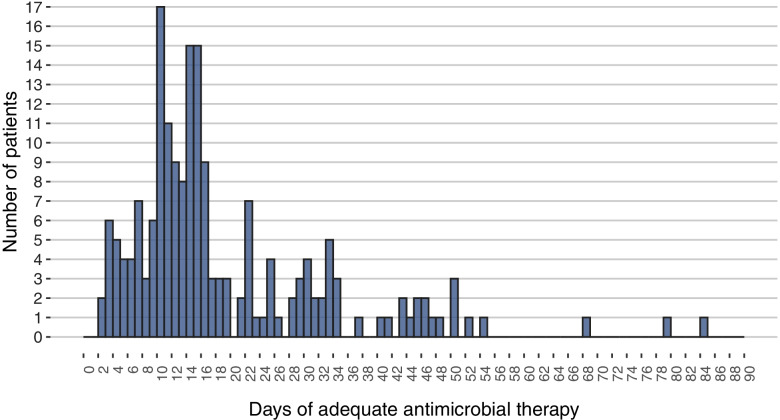
Distribution of treatment durations received by critically ill children with bloodstream infections

Overall, the median duration of adequate antimicrobial treatment was 15 (IQR 11–25) days (Fig. 1). There was wide variability in treatment durations both within and across PICU sites (Supplement Table 1).

### Patient outcomes

Relapses of the underlying focus of infection, defined as recurrence of infection from the date of cessation of antimicrobial therapy to the end of hospital stay were documented for 13 (7%) patients. Overall, 167 (89%) patients were discharged from the PICU alive, 34 (18%) patients required readmission to the PICU and 163 (87%) were discharged alive from hospital.

### Patient characteristics and treatment duration

In univariate analyses, there were no significant differences in the durations of adequate antimicrobial treatment received by patients across age groups, comorbidity profiles, or PRISM-IV severity of illness score (Table 2).

**Table 2:** Patient characteristics and treatment duration

**Characteristic**	***n* (%)**	**Median (days)**	IQR (days)	***p*-value**^***a***^
**Age (years)**				
< 1	104 (56)	15	11–23.5	0.31^*b*^
1–4	41 (22)	17	12–31	
5–11	21 (11)	15	11–20	
≥ 12	21 (11)	15	10–23	
**Severity of illness**				
PRISM-IV^*c*^ score				0.2
< 8	93 (50)	15	11–23	
≥ 8	94 (50)	16	12–30	
**Comorbidities**				
Cardiovascular				0.97
Yes	71 (38)	16	11–25	
No	116 (62)	15	11–25	
Respiratory				0.74
Yes	42 (23)	15.5	12–20	
No	145 (78)	15	11–27	
Neurological				0.94
Yes	24 (13)	16	13.5–19.5	
No	163 (87)	15	11–26	
Immunosuppressed				0.48
Yes	43 (23)	15	10–23	
No	144 (77)	15	11–26	

*IQR* interquartile range

^*a*^Wilcoxon rank sum

^*b*^Kruskal-Wallis test

^*c*^PRISM-IV score dichotomized at overall study cohort median score of 8

### Infection characteristics and treatment duration

**Fig. 2:**
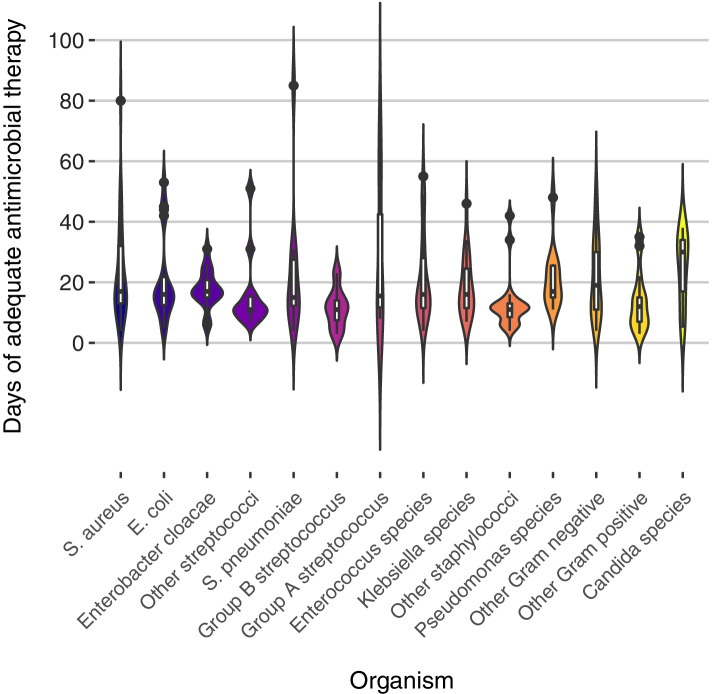
Treatment durations according to pathogens. Boxplots indicating the median, IQR and the upper and lower adjacent values are overlaid within each violin plot. In some cases, more than one treatment duration peak is evident in the violin plots and reflects the multimodal nature of treatment durations used to treat the same pathogen or pathogen group(s). The amplitude of the peaks relative to each other expresses the density of observations seen along the spectrum of treatment durations plotted

The three most common organisms causing infections were *Staphylococcus aureus* (13%), *Enterococcus* species (12%) and *Klebsiella* species (10%). The violin plots in Fig. 2 compare the distribution of treatment durations according to pathogen groups. Across pathogens, the median (IQR) treatment durations were generally similar, with the exception of infections caused by other staphylococci (including coagulase negative staphylococci) which were treated for shorter duration (median 11 (IQR 7–13) days) compared to the rest of the other pathogens. Infections caused by *Candida* species were treated for a median duration of 30 (IQR 17–34) days (Table 3).

**Table 3:** Infection characteristics and treatment duration

Characteristic	*n* (%)	Median (days)	IQR (days)	*p*-value^*a*^
**Pathogen**				
* Staphylococcus aureus*	29 (13)	17	13–32	0.03^*b*^
* Enterococcus* species	27 (12)	16	11–30	
Other staphylococci, including CONS	19 (8)	11	7–13	
* Streptococcus* species				
* S. pneumoniae*	10 (4)	15	12–29	
Group B streptococcus	7 (3)	11	5–15	
Group A streptococcus	6 (3)	15.5	11–51	
Other streptococci	12 (5)	11	9.5–15.5	
* Klebsiella* species	23 (10)	16	11–26	
* Escherichia coli*	18 (8)	16	12–23	
* Enterobacter cloacae*	16 (7)	17	15–21.5	
* Pseudomonas* species	11 (5)	17	15–26	
Other Gram negative bacteria	25 (11)	19	11–30	
Other Gram positive bacteria	13 (6)	12	7–15	
* Candida* species	9 (4)	30	17–34	
**Underlying source**				
Vascular catheter				0.81
Yes	77 (41)	16	11–24	
No	110 (59)	15	11–25	
Respiratory				0.74
Yes	34 (18)	15	11–26	
No	153 (82)	15	11–23	
Intra-abdominal				0.04
Yes	22 (12)	20	14–26	
No	165 (88)	15	11–23	
Skin/soft tissue				0.02
Yes	20 (11)	17	15–33	
No	167 (89)	15	11–23	
Urinary				0.34
Yes	13 (7)	17	15–35	
No	174 (93)	15	11–24	
CNS				0.02
Yes	7 (4)	33	15–46	
No	180 (96)	15	11–23	
Other source(s)^*c*^				0.07
Yes	6 (3)	29.5	15–55	
No	181 (97)	15	11–23	
Unclear source				0.02
Yes	37 (20)	13	10–16	
No	150 (80)	16	11–27	

*IQR* interquartile range, *CONS* coagulase negative staphylococci

^a^Wilcoxon rank sum

^b^Kruskal-Wallis test

^c^Other sources: 3 retropharyngeal abscesses, 1 possibly related to cardiac surgery, 1 endovasculitis, 1 unspecified

**Fig. 3:**
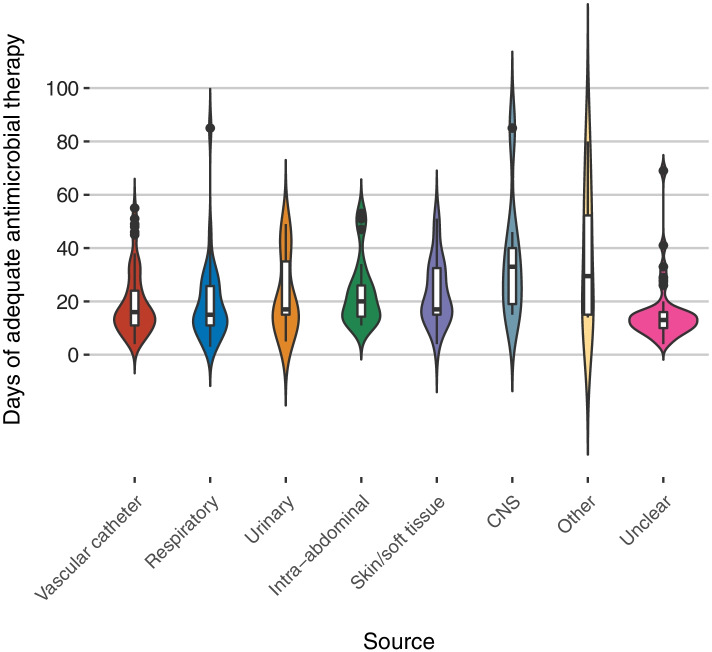
Treatment durations according to underlying sources of infection

In violin plots illustrating the distributions of treatment duration according to underlying sources of BSI, more than one treatment duration peak is also seen in each plot indicating different treatment durations used for the same source(s) of infection (Fig. 3). In univariate analyses, the median treatment durations were significantly longer in patients whose infections were attributed to sources in the abdomen, CNS and skin/soft tissues. In contrast, median treatment duration was shorter when the source of infection was unclear (Table 3).

### Multivariable linear regression analysis

In multivariable linear regression analysis, a CNS source of infection was associated with an additional 17 (95% CI, 6.7–27.4, *p* = 0.001) days of antimicrobial treatment, whereas a source of infection classified as ‘Other’ was associated with an additional 19.2 (95% CI, 7.6–30.8, *p* = 0.001) days, if all other variables were held constant. Infections that were classified as ‘Other’ included three retropharyngeal abscesses, one endovasculitis, one infection possibly related to cardiac surgery and one unspecified infection. With the same regression model adjusted for other variables, treatment duration was also associated with increased patient severity of illness by 0.4 (95% CI, 0.1–0.7, *p* = 0.007) additional days of therapy for each increase in PRISM-IV score by one unit. The other variables: age, comorbidities and pathogen were not associated with the duration of antimicrobial therapy received (Table 4).

**Table 4:** Multivariable patient and pathogen predictors of antimicrobial treatment duration

Predictor	Adjusted beta coefficient	95% CI	*p*-value
Age in years	-0.1	-0.6 to + 0.3	0.62
PRISM-IV score	+ 0.4	+ 0.1 to + 0.7	0.007
Comorbidities			
Cardiovascular	-0.4	-4.7 to + 3.9	0.85
Respiratory	-1.4	-6.4 to + 3.6	0.58
Neurologic	-1.9	-8 to + 4.2	0.54
Immunosuppressed	-2.4	-7.3 to + 2.6	0.34
Pathogen group			
* Staphylococcus aureus*	+ 1.7	-6.2 to + 9.5	0.68
* Enterococcus* species	+ 0.3	-7.1 to + 7.7	0.93
Other staphylococci/CONS	-6.2	-15.4 to + 3	0.19
* Streptococcus* species	-0.7	-7.6 to + 6.3	0.85
Other Gram negative bacteria	+ 2.5	-4.3 to + 9.3	0.47
Other Gram positive bacteria	-8.6	-19.1 to + 1.8	0.11
* Candida* species	+ 5.3	-6.7 to + 17.3	0.38
Polymicrobial	+ 0.4	-7.2 to + 8	0.92
* Enterobacterales*	*reference*	–	–
Underlying source			
Vascular catheter	-1.4	-6.9 to + 4.1	0.62
Respiratory	-1.8	-7.8 to + 4.3	0.57
Urinary	+ 1.2	-7.6 to + 9.9	0.79
Intra-abdominal	+ 4.1	-2.4 to + 10.7	0.22
Skin/soft tissue	+ 0.9	-6.2 to + 8	0.8
CNS	+ 17	+ 6.7 to + 27.4	0.001
Other^*a*^	+ 19.2	+ 7.6 to + 30.8	0.001
Unclear	-4.4	-11.1 to + 2.3	0.2

*CI* confidence interval, *CONS* coagulase negative staphylococci

Number of PICU sites = 6

Mixed model, PICU site included as random effect

^a^Other sources: 3 retropharyngeal abscesses, 1 possibly related to cardiac surgery, 1 endovasculitis, 1 unspecified

### Sensitivity analyses

In sensitivity analyses, the exclusion of patients with CNS infections, patients less than 3 months of age and patients with fungemia did not significantly change the median duration of antimicrobial therapy (Supplement Table 2). Severity of illness (PRISM-IV), CNS infections and ‘Other’ infections remained significantly associated with treatment duration in multivariable linear regression when patients with fungemia were excluded. The results were similar with the exclusion of patients who were less than 3 months of age. When patients with CNS infections were excluded, PRISM-IV and ‘Other’ infections remained significantly associated with longer antimicrobial treatment duration.

The multivariable linear regression model adjusted for PICU site was similar to the model where PICU site was a random effect. Severity of illness (PRISM-IV), CNS infections and ‘Other’ infections remained significantly associated with treatment duration (Supplement Table 3) while PICU site was not significant.

## Discussion

In our observational study of critically ill children with BSIs in six Canadian PICUs, we found that half of these children received at least 15 days of antimicrobial therapy. Treatment durations were similar regardless of patients’ ages or pre-existing comorbidities. Bacteremia secondary to intra-abdominal, skin/soft tissue and CNS infections were treated for longer periods. In contrast, the treatment duration was shorter if the source of infection was unclear. Infections caused by coagulase negative staphylococci (not *S. aureus*) were treated for shorter durations compared to other organisms, while infections caused by *Candida *species were treated for longer. This is not surprising since coagulase negative staphylococci infections are usually less virulent causing less severe infections, while fungemia has been shown to be associated with higher infection-related mortality and potential metastatic complications including chorioretinitis [21]. However, in multivariable linear regression, only CNS infections, infections classified as ‘Other’ and severity of illness were independently associated with longer treatment durations.

Our finding that BSIs in critically ill children are treated for a median of 15 (IQR 11–25) days is comparable to a similar study conducted in critically ill adults who received a median treatment duration of 14 (IQR 9–17.5) days [15]. Since children have different underlying diseases and comorbidities as well as age-related differences in susceptibilities and exposures to different infections compared to adults, we usually expect clinical practices to be different between the management of critically ill children and adults. Nevertheless, our study results, together with those of the adult study [15], reveal that most BSIs are being treated for at least two weeks in both adult and pediatric critically ill patients.

We also observed considerable variability in overall treatment durations both within and between different PICUs across Canada. The practice variations and the lack of substantial differences seen in treatment duration across most patient, pathogen and infection characteristics suggests that uncertainty exists about the appropriate treatment duration for BSIs. Most studies on antimicrobial treatment duration for BSIs in neonates and children have focused on bacteremia related to specific syndromes or pathogens separately, such as meningitis [29, 30], *S. aureus *[31–34] or Gram negative organisms [35, 36]. The resulting evidence has mostly been graded as only sufficient to provide some or weak support for recommendations requiring care or caution if applied to practice [37]. Observational studies, randomized controlled trials and systematic reviews comparing long- versus short-duration antibiotic therapy for bacteremia suggest that shorter treatment courses in neonates and children have similar clinical outcomes to longer treatments [9, 31, 35, 38–40]. Yet, there are also exceptions where longer duration therapy appears to be more appropriate, such as bacteremia caused by *S. aureus* or when indwelling central lines are not removed [31, 35].

The nature of critical care requires clinicians to make clinical decisions amidst evolving patient characteristics, changing clinical parameters, new laboratory findings and fluctuating clinical stability. These complex factors, in addition to differences in institutional culture and the general lack of evidence-based guidelines for treatment duration of infections have also been shown to contribute to significant variations in prescribing practice regarding antimicrobial use [41].

In an era of increasing antimicrobial resistance, it is important to develop strategies to reduce antimicrobial overuse. One way to achieve this would be to optimize the duration of antimicrobial therapy and provide the shortest effective and safe duration of treatment. Our study results provide important data that demonstrates there is a need to study what duration is actually needed for adequate treatment of BSIs in critically ill children and to determine whether shorter treatment durations could be appropriate for patients with certain clinical and infection-related characteristics. More prospective research to produce evidence that will inform and develop optimal prescribing policies for treating BSIs is needed.

Strengths of this study include the range of participating PICUs across five cities in four provinces across Canada, the capture of all systemic antimicrobial therapies received by patients from the first positive index blood culture and adjudication in every case to confirm that the antimicrobial therapies used were reported active against the pathogen when treatment duration was calculated. We also mitigated survivor bias by excluding patients who died early while on antibiotics so that their deaths would not be incorrectly attributed to having received short duration antimicrobial therapy. Our study results are generalizable because we did not put restrictions on the sources of infection or types of pathogen included, with the exception of contaminating organisms and the few foci of infection where prolonged treatment is well-established. We also used multivariable linear regression analysis to account for pre-selected differences between patients, pathogens and infections, as well as clustering of patients within PICUs.

There are some limitations to this study due to the retrospective observational design. Adequate treatment duration was adjudicated based on the days of consecutive therapy with active antimicrobials, but we could not confirm that the antimicrobials were always intended for the treatment of the BSI. The determination of an underlying source of infection in a retrospective chart review was challenging because this was not always explicitly stated in the chart and reported signs and symptoms and test results alone do not always sufficiently clarify the source. Clinical decisions about treatment duration depend on the clinical context and there could have been other confounding factors influencing treatment duration that were not captured. Finally, details about the timing of source control, which would influence the duration of antimicrobial therapy prescribed, were not captured.

## Conclusions

In our retrospective observational study, most critically ill children with BSIs received long courses of antimicrobial therapy lasting at least two weeks and practice heterogeneity was seen both within and across different PICUs. Our findings support a need for further study to determine whether shorter duration therapy could be appropriate for certain patients or infectious syndromes. Optimizing treatment duration has the potential to reduce unnecessary antimicrobial use, complications associated with antimicrobial therapies and development of resistance.

## Supplementary Information

**Additional file 1. **

**Additional file 2. **

**Additional file 3. **

**Additional file 4. **

**Additional file 5. **

## Data Availability

The datasets used and analysed during the current study are available from the corresponding author on reasonable request.

## Abbreviations

BSI: Bloodstream infection
CNS: Central nervous system
CI: Confidence interval
IQR: Interquartile range
PICU: Pediatric intensive care unit
SD: Standard deviation

## References

[1] Laupland KB, Gregson DB, Vanderkooi OG (2009). The changing burden of pediatric bloodstream infections in Calgary, Canada, 2000–2006. Pediatr Infect Dis J.

[2] Spaulding AB, Watson D, Dreyfus J (2019). Epidemiology of bloodstream infections in hospitalized children in the United States, 2009–2016. Clin Infect Dis.

[3] Kumar A, Roberts D, Wood KE (2006). Duration of hypotension before initiation of effective antimicrobial therapy is the critical determinant of survival in human septic shock. Crit Care Med.

[4] Weiss SL, Fitzgerald JC, Balamuth F (2014). Delayed antimicrobial therapy increases mortality and organ dysfunction duration in pediatric sepsis. Crit Care Med.

[5] Seymour CW, Gesten F, Prescott HC (2017). Time to treatment and mortality during mandated emergency care for sepsis. N Engl J Med.

[6] Peltan ID, Brown SM, Bledsoe JR (2019). ED door-to-antibiotic time and long-term mortality in sepsis. Chest.

[7] Liu VX, Fielding-Singh V, Greene JD (2017). The timing of early antibiotics and hospital mortality in sepsis. Am J Respir Crit Care Med.

[8] Evans IVR, Phillips GS, Alpern ER (2018). Association between the New York sepsis care mandate and in-hospital mortality for pediatric sepsis. JAMA.

[9] Havey TC, Fowler RA, Daneman N (2011). Duration of antibiotic therapy for bacteremia: a systematic review and meta-analysis. Crit Care.

[10] Timsit JF, Ruppe E, Barbier F (2020). Bloodstream infections in critically ill patients: an expert statement. Intensive Care Med.

[11] Mermel LA, Allon M, Bouza E (2009). Clinical practice guidelines for the diagnosis and management of intravascular catheter-related infection: 2009 update by the Infectious Diseases Society of America. Clin Infect Dis.

[12] Same RG, Hsu AJ, Tamma PD (2019). Optimizing the management of uncomplicated gram-negative bloodstream infections in children: translating evidence from adults into pediatric practice. J Pediatr Infect Dis.

[13] Spellberg B, Rice LB (2019). Duration of antibiotic therapy: shorter is better. Ann Int Med.

[14] Wald-Dickler N, Spellberg B (2019). Short-course antibiotic therapy–replacing Constantine units with “shorter is better”. Clin Infect Dis.

[15] Daneman N, Rishu AH, Xiong W (2016). Duration of antimicrobial treatment for bacteremia in Canadian critically ill patients. Crit Care Med.

[16] Daneman N, Shore K, Pinto R, Fowler R (2011). Antibiotic treatment duration for bloodstream infections in critically ill patients: a national survey of Canadian infectious diseases and critical care specialists. Int J Antimicrob Agents.

[17] Daneman N, Rishu AH, Xiong W (2015). Bacteremia Antibiotic Length Actually Needed for Clinical Effectiveness (BALANCE): study protocol for a pilot randomized controlled trial. Trials.

[18] Daneman N, Rishu AH, Pinto RL (2020). Bacteremia Antibiotic Length Actually Needed for Clinical Effectiveness (BALANCE) randomised clinical trial: study protocol. BMJ Open.

[19] Yogaraj JS, Elward AM, Fraser VJ (2002). Rate, risk factors and outcomes of nosocomial primary bloodstream infection in pediatric intensive care unit patients. Pediatrics.

[20] Weiner-Lastinger LM, Abner S, Benin AL (2020). Antimicrobial-resistant pathogens associated with pediatric healthcare-associated infections: summary of data reported to the National Healthcare Safety Network, 2015–2017. Infect Control Hosp Epidemiol.

[21] Weiner-Lastinger LM, Abner S, Edwards JR (2020). Antimicrobial-resistant pathogens associated with adult healthcare-associated infections: summary of data reported to the National Healthcare Safety Network, 2015–2017. Infect Control Hosp Epidemiol.

[22] Armenian SH, Singh J, Arrieta AC (2005). Risk factors for mortality resulting from bloodstream infections in a pediatric intensive care unit. Pediatr Infect Dis J.

[23] Lim SY, Pettit RS (2019). Pharmacokinetic considerations in pediatric pharmacotherapy. Am J Health-Syst Pharm.

[24] Kearns GL, Abdel-Rahman SM, Alander SW (2003). Developmental pharmacology–drug disposition, action, and therapy in infants and children. N Engl J Med.

[25] Baddour LM, Wilson WR, Bayer AS (2015). Infective endocarditis in adults: diagnosis, antimicrobial therapy, and management of complications: a scientific statement for healthcare professionals from the American Heart Association. Circulation.

[26] Berbari EF, Kanj SS, Kowalski TJ (2015). 2015 Infectious Diseases Society of America (IDSA) Clinical practice guidelines for the diagnosis and treatment of native vertebral osteomyelitis in adults. Clin Infect Dis.

[27] Solomkin JS, Mazuski JE, Bradley JS (2010). Diagnosis and management of complicated intra-abdominal infection in adults and children: guidelines by the Surgical Infection Society and the Infectious Diseases Society of America. Clin Infect Dis.

[28] Osmon DR, Berbari EF, Berendt AR (2013). Diagnosis and management of prosthetic joint infection: clinical practice guidelines by the Infectious Diseases Society of America. Clin Infect Dis.

[29] Molyneux E, Nizami SQ, Saha S (2011). 5 versus 10 days of treatment with ceftriaxone for bacterial meningitis in children: a double-blind randomised equivalence study. Lancet.

[30] Roine I, Ledermann W, Foncea LM (2000). Randomized trial of four vs. seven days of ceftriaxone treatment for bacterial meningitis in children with rapid initial recovery. Pediatr Infect Dis J.

[31] Chowdhary G, Dutta S, Narang A (2006). Randomized controlled trial of 7-day vs. 14-day antibiotics for neonatal sepsis. J Trop Pediatr.

[32] Walker TM, Bowler ICJW, Bejon P (2009). Risk factors for recurrence after Staphylococcus aureus bacteremia–a retrospective matched case-control study. J Infect.

[33] Creel AM, Durham SH, Benner KW (2009). Severe invasive community-associated methicillin-resistant Staphylococcus aureus infections in previously healthy children. Pediatr Crit Care Med.

[34] Liu C, Bayer A, Cosgrove SE (2011). Clinical practice guidelines by the Infectious Diseases Society of America for the treatment of methicillin-resistant Staphylococcus aureus infections in adults and children. Clin Infect Dis.

[35] Park SH, Milstone AM, Diener-West M (2014). Short versus prolonged courses of antibiotic therapy for children with uncomplicated gram-negative bacteraemia. J Antimicrob Chemother.

[36] Tsai MH, Huang YC, Chiu CH (2007). Nontyphoidal Salmonella bacteremia in previously healthy children: analysis of 199 episodes. Pediatr Infect Dis J.

[37] McMullan BJ, Andresen D, Blyth CC (2016). Antibiotic duration and timing of the switch from intravenous to oral route for bacterial infections in children: systematic review and guidelines. Lancet Infect Dis.

[38] Gathwala G, Sindwani A, Singh J (2010). Ten days vs. 14 days antibiotic therapy in culture-proven neonatal sepsis. J Trop Pediatr.

[39] Onakpoya IJ, Walker AS, Tan PS (2018). Overview of systematic reviews assessing the evidence for shorter versus longer duration antibiotic treatment for bacterial infections in secondary care. PLoS one.

[40] Rohatgi S, Dewan P, Faridi MMA (2017). Seven versus 10 days antibiotic therapy for culture-proven neonatal sepsis: a randomised controlled trial. J Paediatr Child Health.

[41] Noel KC, Papenburg J, Lacroix J (2020). International survey on determinants of antibiotic duration and discontinuation in pediatric critically ill patients. Pediatr Crit Care Med.

