# Supplementary material for: Antimicrobial treatment duration for uncomplicated bloodstream infections in critically ill children: a multicentre observational study
Source: BMC Pediatr. 2022 Apr 5;22:179. doi: 10.1186/s12887-022-03219-z (PMC8981828; doi:10.1186/s12887-022-03219-z)
Supplement: Supplementary file 1 — Additional file 1. [file 12887_2022_3219_MOESM1_ESM.docx]

**Supplement Table 1.** Variation in duration of adequate antimicrobial treatment within and across pediatric intensive care units

| Site | n (%) | Median (days) | IQR (days) |
| --- | --- | --- | --- |
| Overall | 187 (100) | 15 | 11-25 |
| A | 72 (39) | 16 | 13-26 |
| B | 35 (19) | 12 | 11-31 |
| C | 32 (17) | 13.5 | 8-19.5 |
| D | 21 (11) | 22 | 13-35 |
| E | 16 (9) | 11.5 | 10.5-16 |
| F | 11 (6) | 22 | 10-31 |

IQR = interquartile range
