# Supplementary material for: Antimicrobial treatment duration for uncomplicated bloodstream infections in critically ill children: a multicentre observational study
Source: BMC Pediatr. 2022 Apr 5;22:179. doi: 10.1186/s12887-022-03219-z (PMC8981828; doi:10.1186/s12887-022-03219-z)
Supplement: Supplementary file 2 — Additional file 2. [file 12887_2022_3219_MOESM2_ESM.docx]

**Supplement Table 2.** Duration of adequate antimicrobial treatment

| Patients | Number of patients | Median (days) | IQR (days) |
| --- | --- | --- | --- |
| Overall | 187 | 15 | 11-25 |
| Exclude patients with fungemia | 178 | 15 | 11-23 |
| Exclude CNS infections | 180 | 15 | 11-23 |
| Exclude patients <3 months old | 146 | 16 | 11-26 |
