# Supplementary material for: Antimicrobial treatment duration for uncomplicated bloodstream infections in critically ill children: a multicentre observational study
Source: BMC Pediatr. 2022 Apr 5;22:179. doi: 10.1186/s12887-022-03219-z (PMC8981828; doi:10.1186/s12887-022-03219-z)
Supplement: Supplementary file 3 — Additional file 3. [file 12887_2022_3219_MOESM3_ESM.docx]

**Supplement Table 3.** Multivariable patient and pathogen predictors of antimicrobial treatment duration adjusted for pediatric intensive care unit site

| Predictor | Adjusted beta coefficient | 95% CI | p-value |
| --- | --- | --- | --- |
| Age in years | -0.1 | -0.5 to +0.4 | 0.69 |
| PRISM-IV score | +0.4 | +0.1 to +0.7 | 0.006 |
| Comorbidities  Cardiovascular  Respiratory  Neurologic  Immunosuppressed | -0.8  -1.9  -1.1  -1.9 | -5.3 to +3.6  -7 to +3.2  -7.4 to +5.3  -7 to +3.2 | 0.71  0.47  0.75  0.46 |
| Pathogen group  *Staphylococcus aureus*  *Enterococcus* species  Other staphylococci/CONS  *Streptococcus* species  Other Gram negative bacteria  Other Gram positive bacteria  *Candida* species  Polymicrobial  *Enterobacterales* | +3.2  +0.4  -3.8  -0.004  +2.9  -7.8  +7.1  +1.2  *reference* | -4.8 to +11.2  -7 to +7.8  -13.3 to +5.7  -7 to +7  -3.9 to +9.8  -18.5 to +2.9  -5.1 to +19.4  -6.6 to +9  -- | 0.43  0.91  0.43  0.999  0.4  0.15  0.25  0.76  -- |
| Underlying source  Vascular catheter  Respiratory  Urinary  Intra-abdominal  Skin/soft tissue  CNS  Other*^a^*  Unclear | -1.3  -0.3  +2.5  +4.2  +0.9  +16.3  +17.9  -2.7 | -7.3 to +4.7  -6.7 to +6  -6.6 to +11.7  -2.5 to +10.8  -6.4 to +8.2  +5.7 to +27  +6 to +29.7  -10.2 to +4.7 | 0.67  0.92  0.58  0.22  0.81  0.003  0.003  0.47 |
| Site  F  E  C  B  A  D | -2.6  -8.5  -8  -6  -3.3  *reference* | -13.9 to +8.7  -17.9 to +0.9  -16.2 to +0.3  -13.6 to +1.6  -10.5 to +3.8  -- | 0.65  0.08  0.06  0.12  0.36  -- |

Adjusted R-squared = 0.13

CI = confidence interval, CONS = coagulase negative staphylococci

*^a^*Other sources: 3 retropharyngeal abscesses, 1 possibly related to cardiac surgery, 1 endovasculitis, 1 unspecified
