# Supplementary material for: Antimicrobial treatment duration for uncomplicated bloodstream infections in critically ill children: a multicentre observational study
Source: BMC Pediatr. 2022 Apr 5;22:179. doi: 10.1186/s12887-022-03219-z (PMC8981828; doi:10.1186/s12887-022-03219-z)
Supplement: Supplementary file 4 — Additional file 4. [file 12887_2022_3219_MOESM4_ESM.docx]

**Supplement Table 4.** Multivariable patient and pathogen predictors of antimicrobial treatment duration adjusted for PELOD-2 (day 3) in place of PRISM-IV

| Predictor | Adjusted beta coefficient | 95% CI | p-value |
| --- | --- | --- | --- |
| Age in years | -0.1 | -0.5 to 0.4 | 0.82 |
| PELOD-2, day 3 | 0.6 | -0.1 to 1.2 | 0.1 |
| Comorbidities  Cardiovascular  Respiratory  Neurologic  Immunosuppressed | -0.5  -2.1  -2.4  -2.4 | -4.9 to 3.9  -7.3 to 3.1  -8.6 to 3.8  -7.4 to 2.6 | 0.82  0.42  0.44  0.34 |
| Pathogen group  *Staphylococcus aureus*  *Enterococcus* species  Other staphylococci/CONS  *Streptococcus* species  Other Gram negative bacteria  Other Gram positive bacteria  *Candida* species  Polymicrobial  *Enterobacterales* | 1.9  0.2  -5.9  -0.3  2  -8.7  5.1  1.5  *reference* | -6 to 9.9  -7.3 to 7.7  -15.2 to 3.4  -7.3 to 6.7  -5 to 8.9  -19.3 to 1.9  -7.2 to 17.5  -6.1 to 9.2  -- | 0.63  0.96  0.21  0.93  0.58  0.11  0.42  0.7  -- |
| Underlying source  Vascular catheter  Respiratory  Urinary  Intra-abdominal  Skin/soft tissue  CNS  Other*^a^*  Unclear | -0.4  -1.9  1.1  3.2  1.4  14.8  18.6  -3.5 | -6 to 5.1  -8 to 4.2  -7.8 to 9.9  -3.5 to 9.8  -5.7 to 8.6  4.1 to 25.4  6.8 to 30.4  -10.2 to 3.3 | 0.88  0.54  0.81  0.35  0.69  0.01  0.002  0.32 |

CI = confidence interval, CONS = coagulase negative staphylococci

Number of PICU sites = 6

Mixed model, PICU site included as random effect

*^a^*Other sources: 3 retropharyngeal abscesses, 1 possibly related to cardiac surgery, 1 endovasculitis, 1 unspecified
