# Supplementary material for: Antimicrobial treatment duration for uncomplicated bloodstream infections in critically ill children: a multicentre observational study
Source: BMC Pediatr. 2022 Apr 5;22:179. doi: 10.1186/s12887-022-03219-z (PMC8981828; doi:10.1186/s12887-022-03219-z)
Supplement: Supplementary file 5 — Additional file 5. [file 12887_2022_3219_MOESM5_ESM.docx]

**Supplement Table 5.** Multivariable patient and pathogen predictors of antimicrobial treatment duration adjusted for PELOD-2 (day 4) in place of PRISM-IV

| Predictor | Adjusted beta coefficient | 95% CI | p-value |
| --- | --- | --- | --- |
| Age in years | -0.04 | -0.5 to 0.4 | 0.85 |
| PELOD-2, day 4 | 0.4 | -0.2 to 1.1 | 0.21 |
| Comorbidities  Cardiovascular  Respiratory  Neurologic  Immunosuppressed | -0.6  -2  -2.6  -2.1 | -5 to 3.9  -7.2 to 3.3  -8.8 to 3.5  -7 to 2.9 | 0.8  0.46  0.4  0.42 |
| Pathogen group  *Staphylococcus aureus*  *Enterococcus* species  Other staphylococci/CONS  *Streptococcus* species  Other Gram negative bacteria  Other Gram positive bacteria  *Candida* species  Polymicrobial  *Enterobacterales* | 1.9  0.6  -5.6  -0.2  2.4  -9.3  5.7  1.7  *reference* | -6.1 to 9.9  -6.9 to 8.1  -15 to 3.8  -7.3 to 6.8  -4.5 to 9.3  -20 to 1.4  -6.7 to 18.1  -6 to 9.3  -- | 0.64  0.88  0.24  0.95  0.5  0.09  0.37  0.67  -- |
| Underlying source  Vascular catheter  Respiratory  Urinary  Intra-abdominal  Skin/soft tissue  CNS  Other*^a^*  Unclear | -0.8  -2.1  1  3.1  1.4  15.5  19.2  -3.8 | -6.4 to 4.7  -8.2 to 4  -7.9 to 9.9  -3.6 to 9.7  -5.8 to 8.6  4.9 to 26.1  7.4 to 31  -10.6 to 3 | 0.77  0.49  0.82  0.37  0.7  0.004  0.002  0.27 |

CI = confidence interval, CONS = coagulase negative staphylococci

Number of PICU sites = 6

Mixed model, PICU site included as random effect

*^a^*Other sources: 3 retropharyngeal abscesses, 1 possibly related to cardiac surgery, 1 endovasculitis, 1 unspecified
